# Important Fact

**Published:** 1878-05

**Authors:** 


"I, John Lubberlie, was supposed to be in the last stage of consumption in the year 'forty-eight, suffering at the same time under a severe attack of rheumatism, liver complaint, dropsy, gravel, and cholera morbus. Simultaneously, also, I took the yellow fever, bilious colic, and small pox; the latter assuming the chronic form of scrofula, completely destroying my lungs, liver, spinal marrow, nervous system, and the entire contents of my phrenology. I finally got so low that I did not know my brother-in-law when he came to borrow money. For three months I swallowed nothing but twenty packages of Kunkelhausen's pills, which effected an immediate cure in three weeks.

"My uncle, Bacchus Pottinger, was afflicted so long with the gout that his life became a burden to him. He took only four boxes of said pills and life was a burden to him no longer. Further deponent saith not.

"Sworn and subscribed to, &c., &c."

Or if the patient happened at that critical time to go to the South, or North, or do any extraordinary thing, or any silly thing, such as drinking mule's milk, or goat's cream, or tar water, or brandy smash; if he had slept in a pig-pen, or cow-house, or inhaled hot water, or cold alcohol, or any thing else, the thing last done, has the credit of cure; and thus it is, that although the very next person who "tried" the same remedy died under it, the report has gone abroad, and like the cork leg, couldn't stop itself, and is going yet. Thus the world is full of cures, and any man you meet can deliver at sight half a dozen, any one of which cured a friend of his who was a great deal worse than you are. But to the crushing disappointment of multitudes, the experience is sadly uniform that "whatever it may have done for others, it has not availed for me."

If there be two or more patches of these tubercles, another softens, as the causes of softening are applied, and the same routine is gone through, perhaps until the dozenth time, which being the last, he may live on, to die many years after, of some totally different disease; or if the constitution be not strong, the man succumbs under these repeated attacks, and passes away.

The practical uses to be made of this narration of undoubted facts are various and important: first, it is useless to take any thing without the advice of a regular physician, who must be acquainted with every constituent of the remedy, so as to know in what direction its curative agencies tend; second, do nothing which common sense, joined with professional science, does not indicate as rational and wise.

The reason of these inferences is, the wide difference between an antecedence and subsequence and cause and effect. It is clearly irrational to adopt any remedy, simply because it was applied and restoration followed its application. The scientific practitioner takes no such grounds. It is not until after repeated experiments, made under every variety of circumstances, extending through months, and seasons, and years, giving a uniform result, that he lays hold of any remedy, but once laid hold of, he never rejects it for a single nor for a dozenth failure. Such is the difference between scientific medicine and quackery, between intellect and ignorance. It is only after many a long year's trial, that the skilful practitioner can be brought to say of any remedy, "I gave this, and it cured him." The charlatan speaks thus after the first trial, his ignorance sustaining his effrontery.

Hope is the highest remedy of the soul, the most efficient for the body. This Cluster Doctrine is a true groundwork for it, in consumptive disease. Surely it is Nature's remedy, for who among a thousand does not hope to the end, in consumption? The mischief lies in not making that hopefulness the ground of practical action. The consumptive hopes but does nothing, and thus it is that by hope he lives and dies.

Some of the best medical minds in the world, men who have spent a quarter of a century in examining the lungs of the dead, state to us this important, every day fact, that few people die, after forty, who have not in the lungs, the signs of having the consumption, without ever having had the slightest suspicion of the existence of the disease, and who finally died of maladies having no approximation towards it in nature. These signs, are scars of various lengths, little excavations, or cavities or puckerings of various sizes; all very small it is true, but still showing the great fact, that decay once existed there, and that the lungs may perfectly heal after having been divided or broken, or pierced, as numerous cases bear witness in the perfect recovery of men who have been stabbed in the breast, or shot through the lungs.

